# Function of mitochondrial cytochrome c oxidase is enhanced in human lens epithelial cells at high temperatures

**DOI:** 10.3892/mmr.2022.12906

**Published:** 2022-12-02

**Authors:** Shun Takeda, Naoki Yamamoto, Noriaki Nagai, Noriko Hiramatsu, Saori Deguchi, Natsuko Hatsusaka, Eri Kubo, Hiroshi Sasaki

**Affiliations:** 1 Division for Vision Research, Kanazawa Medical University Graduate School of Medical Science, Kahoku, Ishikawa 920-0293, Japan; 2 Department of Ophthalmology, Kanazawa Medical University, Kahoku, Ishikawa 920-0293, Japan; 3 Division of Vision Research for Environmental Health, Medical Research Institute, Project Research Center, Kanazawa Medical University, Kahoku, Ishikawa 920-0293, Japan; 4 Support Office for Bioresource Research, Research Promotion Headquarters, Fujita Health University, Toyoake, Aichi 470-1192, Japan; 5 International Center for Cell and Gene Therapy, Research Promotion Headquarters, Fujita Health University, Toyoake, Aichi 470-1192, Japan; 6 Faculty of Pharmacy, Kindai University, Higashi-Osaka, Osaka 577-8502, Japan

**Keywords:** high-temperature culture, human, lens epithelium cell, cytochrome c oxidase, ATP, cell proliferation

## Abstract

Enhancement of density via human lens epithelium (HLE) cell proliferation is the underlying cause of nuclear cataracts. Moreover, our previous epidemiological study demonstrated that the risk of nuclear cataract development is significantly higher under elevated environmental temperatures compared with under lower temperatures. The present study investigated the relationship between temperature and cell proliferation in terms of mitochondrial function, which is a nuclear cataract-inducing risk factor, using two different HLE cell lines, SRA01/04 and immortalized human lens epithelial cells NY2 (iHLEC-NY2). Cell proliferation was significantly enhanced under the high-temperature condition (37.5°C) in both cell lines. The cell growth levels of SRA01/04 and iHLEC-NY2 cells cultured at 37.5°C were 1.20- and 1.16-fold those in the low-temperature cultures (35.0°C), respectively. Moreover, the levels of cytochrome c oxidase mRNA (mitochondrial genome, cytochrome c oxidase-1-3) and its activity in SRA01/04 and iHLEC-NY2 cells cultured at 37.5°C were higher compared with those in cells cultured at 35.0°C. In addition, adenosine-5′-triphosphate (ATP) levels in SRA01/04 and iHLEC-NY2 cells were also significantly higher at 37.5°C compared with those at 35.0°C. By contrast, no significant differences in Na^+^/K^+^-ATPase or Ca^2+^-ATPase activities were observed between HLE cells cultured at 35.0 and 37.5°C. These results suggested that expression of the mitochondrial genome was enhanced in high-temperature culture, resulting in a sufficient ATP content and cell proliferation for lens opacity. Therefore, elevated environmental temperatures may increase the risk of nuclear cataracts caused by HLE cell proliferation via mitochondrial activation.

## Introduction

Cataracts are a major cause of visual impairment according to the World Health Organization, second only to uncorrected refractive errors (1). Cataractogenesis is a multifactorial process caused by aggregation of misfolded crystalline proteins (2); cataract types are classified according to the section of the lens exhibiting opacity, such as cortical, nuclear, and posterior subcapsular cataracts. Several factors including aging, diabetes, ultraviolet rays, diet, genetic predisposition, dehydration, oxidative stress, and lipid peroxidation have been shown to cause cataract formation (3). A study of the global burden of diseases reported that cataract treatments are lacking in developing countries and that cataract was the leading cause of blindness in 2020. There are 15.2 million patients with cataract aged 50 years and above, accounting for 45.2% of patients who are blind (4).

Nuclear cataracts typically develop gradually with age and are the most common morphological form of age-related cataracts worldwide (5,6). These comprise the most important category of senile cataract types, as they cause visual disturbances with increasing higher-order aberration, forward light scattering, and backward light scattering even in the early stage (7). Individuals living in geographic regions with higher temperatures tend to develop presbyopia earlier (8). Moreover, our epidemiological studies showed that the prevalence of nuclear cataracts of grade 1 and above as per the World Health Organization cataract grading system was significantly higher in tropical or subtropical regions as compared to that in temperate or subarctic regions, regardless of race (9,10). In our previous research, using a large-scale integrated computational method, we found that the eye lens temperature may reach 37.0-37.5°C or higher with increasing environmental temperature. By setting the threshold of the lens temperature at 37.0°C, we found a positive correlation between the cumulative heat load, which is an indicator of the cumulative temperature difference above 37°C and nuclear cataract incidence for 10 years; we also found an association between the time course of the mean lens temperature rise and nuclear cataract incidence (11). Thus, long-term exposure to a high-temperature environment may increase the risk of nuclear cataract development. However, the detailed association between temperature and nuclear cataracts remains unclear.

A previous study of the human eye showed that the mean nuclear diameter and thickness were 6.51 ± 0.75 and 2.96 ± 0.33 mm, respectively (12) and that these values significantly increased with increasing opacification of the nucleus. Particularly, the thickness and diameter based on the Lens Opacities Classification System III were grades 5 and 6 (12). Moreover, the density of human lens epithelium (HLE) cells of the central anterior capsule in nuclear cataracts was higher than that in normal lenses (>NIII), indicating that individual differences in HLE cell proliferation and density are important underlying causes of nuclear cataracts (13).

The mitochondria play key roles in regulating cell proliferation and apoptosis (14). They also provide energy for anabolic processes, hormone secretion, muscular work, and ionic pumps (15–17). Most of this energy is derived from oxidative phosphorylation, during which electrons are transferred from nicotinamide adenine dinucleotide to oxygen, protons are extruded, and the energy stored as an inner membrane potential is dissipated and accumulated as ATP by ATP synthase. Cytochrome c oxidase (CCO) and sequential oxidoreductive reactions play central roles in ATP production. Specifically, the inner membrane of mitochondria contains protein complexes of the respiratory chain electron transfer system, and electrons pass through complexes I, III and IV (CCO) to oxygen. In conjugation with this electron transfer (redox) reaction, protons (hydrogen ions) are pumped from the inner to the outer membrane, forming a proton electrochemical gradient across the membrane. This potential energy drives the rotational motion of complex V (ATP synthase), enabling the synthesis of ATP. In eukaryotes, the oligomeric enzyme is bound to the mitochondrial inner membrane with 7–13 subunits. Thus, its biosynthesis involves coordinate interplay between the nuclear and mitochondrial genomes. The largest subunits I, II and III, which represent the catalytic core of the enzyme, are encoded by mitochondrial DNA and are synthesized within the mitochondria. The remaining smaller subunits implicated in the regulatory function are encoded by nuclear DNA and imported to the mitochondria following their synthesis in the cytosol. Some nuclear coded subunits are expressed in tissue- and developmental-specific isologs (18). On the other hand, complex IV consists of enzymes composed of cytochrome a, Cu_A_, Cu_B_, and cytochrome a_3,_ and the three subtypes of mitochondrial cytochrome c oxidase 1, 2 and 3 (CCO1-3) relate this CCO function (19). However, it is not clear enough which part of complex IV is composed of CCO-1, 2 and 3.

We previously investigated whether mRNA expression and ATP levels of the three subtypes of mitochondrial cytochrome c oxidase 1, 2 and 3 vary with cataract type and severity in the human lens. We found that mitochondrial CCO1-3 mRNA expression in patients with early-stage cataract was significantly upregulated compared to that in normal patients, indicating that ATP production by the lens epithelium is enhanced in early-stage cataracts in Japanese patients (20). Increased ATP in mitochondria may contribute to lens opacity, making it important to investigate changes in cell proliferation and mitochondrial function in HLE cells under low- and high-temperature conditions to determine the risk of nuclear cataract development in high-temperature environments. In this study, we investigated whether mitochondrial gene expression and function, ATP production, and cell proliferation capacity were altered under low- and high-temperature conditions using two different HLE cell lines, SRA01/04 and iHLEC-NY2.

## Materials and methods

### Reagents

The mitochondrial isolation kit, CCO assay kit, and ATP bioluminescent assay kit were obtained from Sigma-Aldrich (St. Louis, MO, USA). RNase-free DNase and RNeasy Mini kits were provided by Qiagen (Hilden, Germany). The RNA PCR kit was purchased from Takara Bio (Shiga, Japan), and the LightCycler FastStart DNA Master SYBR-Green I was provided by Roche Diagnostics Applied Science (Mannheim, Germany). The Bio-Rad Protein Assay Kit was purchased from Bio-Rad Laboratories (Hercules, CA, USA). All other chemicals used were of the highest commercially available purity.

### Cell lines

The HLE-immortalized cell lines SRA01/04 (RCB1591, Riken BRC, Tsukuba, Japan) and iHLEC-NY2 were donated by Professor Ibaraki (21) and Professor Yamamoto (22). Briefly, iHLEC-NY2 cells were derived from lens epithelial cells (LECs) collected during cataract surgery in adult humans. LECs were cultured in Dulbecco's modified Eagle's medium (DMEM) high-glucose medium containing 10% fetal bovine serum (FBS). The immortalization gene was transferred to the pseudo-attachment P site of cultured LECs, and the cells were cloned to establish the immortalized human lens epithelial cell line clone NY2 (iHLEC-NY2) (22). The use of iHLEC-NY2 cells was approved by the Fujita Medical University Recombinant DNA Experiment Safety Committee (approval no. DP16055) and Kanazawa Medical University Recombinant DNA Experiment Safety Committee (approval no. 2020-18). Mycoplasma infections in SRA01/04 and iHLEC-NY2 cells were detected using a Mycoplasma Detection Kit (EZ-PCRTM Mycoplasma Test Kit, Biological Industries, Beit Haemek, Israel) according to the manufacturer's instructions.

### Cell culture

SRA01/04 cells were cultured in-low glucose DMEM (Thermo Fisher Scientific, Waltham, MA, USA) containing 20% (v/v) heat-inactivated and γ-ray-sterilized FBS (Biological Industries) and penicillin-streptomycin solution (FUJIFILM Wako Pure Chemical Corporation, Osaka, Japan). iHLEC-NY2 cells were cultured in high-glucose DMEM containing 10% FBS, 10 ng/ml basic fibroblast growth factor, 100× minimum essential medium non-essential amino acids, 100× GlutaMAX™ (Thermo Fisher Scientific), and penicillin-streptomycin solution. In computer simulations, when the ambient temperature was 19–35°C, the estimated lens temperature was 35.0-37.5°C (11). We previously reported that the prevalence of nuclear cataracts is significantly higher in tropical regions than in temperate regions (9,10,23). The lens epithelial cell density and proliferation capacity *in vivo* may be important underlying causes of cataracts at the cellular level, particularly in nuclear cataracts (13). Therefore, both SRA01/04 and iHLEC-NY2 cells were incubated under humidified air containing 5% CO_2_ at 35.0°C or 37.5°C.

### Measurement of cell proliferation

The cells were seeded at a density of 2.5×10^4^ cells per well in a cell culture dish (60-mm diameter, Eppendorf, Hamburg, Germany) and cultured for four days at 35.0°C or 37.5°C, with the medium replaced every other day. Cell growth was measured using a Cell Counting Kit-8 (WST-8, FUJIFILM Wako Pure Chemical Corporation), and the absorbance was measured at 450 nm in a Benchmark Microplate Reader (Bio-Rad Laboratories).

### Measurement of cell apoptosis

SRA01/04 and iHLEC-NY2 cells cultured at 35.0 and 37.5°C were removed from the culture dishes and washed with phosphate-buffered saline. Since iHLEC-NY2 is a GFP-expressing cell, SRA01/04 and iHLEC-NY2 cells were stained with Annexin V-APC antibody and Propidium iodide (APC-conjugated Annexin V Apoptosis Detection Kit, Biolegend, San Diego, CA, USA), and were analyzed by flow cytometry (CytoFLEX^TM^ Flow Cytometer, Beckman Coulter, Inc., Brea, CA, USA).

### Quantitative real-time RT-PCR

mRNA expression was measured using a LightCycler DX 400, according to our previous report (20). Briefly, total RNA was extracted from the samples and purified using an RNase-Free DNase Set and RNeasy Mini Kit. Reverse transcription was performed using an RNA PCR kit, and PCR was performed using the LightCycler FastStart DNA Master SYBR-Green I. The RT reaction was carried out at 42°C for 15 min, followed by 95°C for 5 min. The PCR conditions were as follows: 95°C for 10 min (hot start); 60 cycles at 95°C for 10 sec (denaturing), 63°C for 10 sec (annealing), and 72°C for 5 sec (extension). The following specific primers (final concentration 10 pmol) were used: 5′-CCGTCCTAATCACAGCAGTCCTA-3′ and 5′-TGAGGTTGCGGTCTGTTAGTAGT-3′ for CCO-1 (gene ID: 4512, accession nos.: NC_012920.1, coding sequence: YP_003024028.1, primer position: 6463-6549); 5′-CCGCCATCATCCTAGTCCTCAT-3′ and 5′-GATCGTTGACCTCGTCTGTTATGT-3′ for CCO-2 (gene ID: 4513, accession nos.: NC_012920.1, coding sequence: YP_003024029.1, primer position: 7791-7862); 5′-ACGGCATCTACGGCTCAACA-3′ and 5′-TGGCGGATGAAGCAGATAGTGA-3′ for CCO-3 (gene ID: 4514, accession nos.: NC_012920.1, coding sequence: YP_003024032.1, primer position: 9775-9871); and 5′-TGCACCACCAACTGCTTAGC-3′ and 5′-GGCATGGACTGTGGTCATGAG-3′ for glyceraldehyde-3-phosphate dehydrogenase (gene ID: 2597, accession nos.: NM_002046.7, coding sequence: NP_002037.2, primer position: 530-616). The differences in the threshold cycles between the target groups (CCO-1, CCO-2, and CCO-3) and GAPDH were used to calculate the mRNA expression levels in the samples.

### Measurement of protein

The protein levels in the samples were determined using the Bradford method with the Bio-Rad Protein Assay Kit with bovine serum albumin as the standard.

### Measurement of CCO activity in mitochondria

Mitochondria were isolated using a mitochondrial isolation kit, and CCO activity was measured using a CCO assay kit according to a previous report (24). Briefly, HLE cells were washed with ice-cold phosphate-buffered saline, homogenized in pH 7.5 isolation buffer, and centrifuged at 600 × g for 5 min at 4°C. The supernatants were centrifuged at 11,000 × g for 10 min at 4°C, and the pellets were suspended in isolation buffer. The samples were further centrifuged at 600 × g for 5 min at 4°C, and the supernatants were centrifuged at 11,000 × g for 10 min at 4°C. The isolated mitochondria were added to pH 7 buffer consisting of 10 mM Tris-HCl, 120 mM KCl, and 250 mM sucrose, and the reaction was initiated by adding ferrocytochrome c (reduced with 0.1 M dithiothreitol). The decrease in absorbance at 550 nm was measured for 1 min using a UV2200 spectrophotometer (Shimadzu Corporation, Kyoto, Japan) according to the manufacturer's instructions. CCO activity was determined from the decrease in the level of absorbance at 550 nm and expressed as min/mg protein.

### Measurement of ATP

ATP was measured using the luciferin-luciferase assay method as described previously (18,24,25). Samples were homogenized in 100 µl saline and centrifuged at 20,400 × g for 15 min at 4°C. The resulting supernatant was assayed using an ATP bioluminescent assay kit and luminometer AB-2200 (Atto Corporation, Tokyo, Japan). ATP levels were expressed as nmol/mg protein.

### Measurement of ATPase activity

Both Ca^2+^-ATPase and Na^+^/K^+^-ATPase activities were measured as previously described (26). Briefly, HLE cells were washed with ice-cold pH 7.4 Ca^2+^, Mg^2+^-free buffer (290 mOsm) and harvested using a cell scraper (Iwaki Co., Ltd., Tokyo, Japan). The composition of the pH 7.4 Ca^2+^, Mg^2+^-free buffer was as follows: 0.5 mM EDTA, 5 mM NaHCO_3_, 5 mM KCl, 8 mM Tris, 15 mM HEPES, and 145 mM NaCl in water. The collected cells were homogenized in 600 ml of hypotonic buffer (pH 7.4) consisting of 10 mM mannitol, 5.75 mM HEPES, and 6.25 mM Tris base. Unbroken cells were pelleted at a low speed (2,040 × g, 10 min, 4°C), and the supernatant obtained was assayed for ATPase activity (assessed as Pi liberated from ATP). Ca^2+^-ATPase activity was calculated as the difference in phosphate release measured in the presence or absence of 0.1 mM Ca^2+^. Na^+^/K^+^-ATPase activity was calculated as the difference in phosphate release measured in the presence or absence of 1 mM ouabain. The activity of both plasma membrane Ca^2+^-ATPase (PMCA) and sarco (endo) plasmic reticulum Ca^2+^-ATPase (SERCA) was measured as Ca^2+^-ATPase activity in this study.

### Statistical analysis

The data are expressed as the mean ± standard error, and statistical analysis was performed using unpaired Student's t-test. The significance level was set at P<0.05.

## Results

### Effect of high-temperature culture on growth of SRA01/04 and iHLEC-NY2 cells

**Figure 1.**
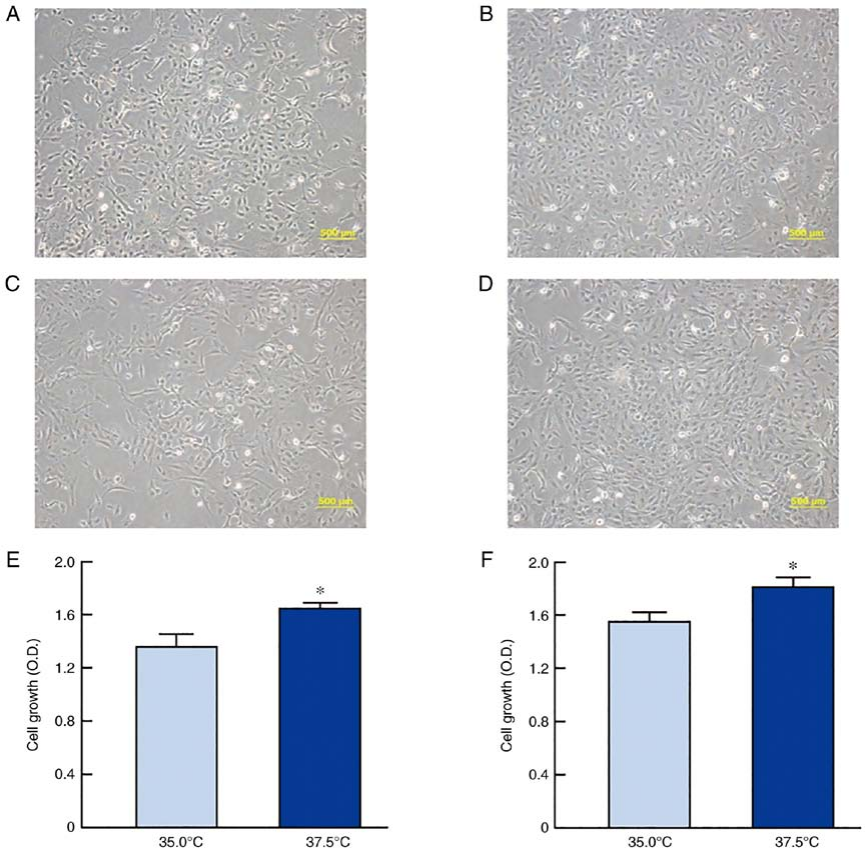
Cell morphology of SRA01/04 cells at (A) 35.0°C and (B) 37.5°C. Cell morphology of iHLEC-NY2 cells at (C) 35.0°C and (D) 37.5°C (scale bar, 500 µm). Cell proliferation of (E) SRA01/04 cells and (F) iHLEC-NY2 cells at 35.0°C or 37.5°C. n=5. *P<0.05 vs. 35.0°C. iHLEC-NY2, immortalized human lens epithelial cells NY2; OD, optical density.

The relationship between temperature and cell proliferation was determined using two HLE cell lines (SRA01/04 and iHLEC-NY2). Fig. 1 shows the changes in the cell morphology and proliferation of SRA01/04 and iHLEC-NY2 cells under high- or low-temperature culture. Cell proliferation was significantly enhanced at high temperature for both SRA01/04 and iHLEC-NY2 cells, and the growth levels of SRA01/04 and iHLEC-NY2 cells cultured at 37.5°C were 1.20- and 1.16-fold higher than those at 35.0°C (P<0.05), respectively.

### Activation of mitochondrial function (CCO activity) in SRA01/04 and iHLEC-NY2 cells under high-temperature culture

**Figure 2.**
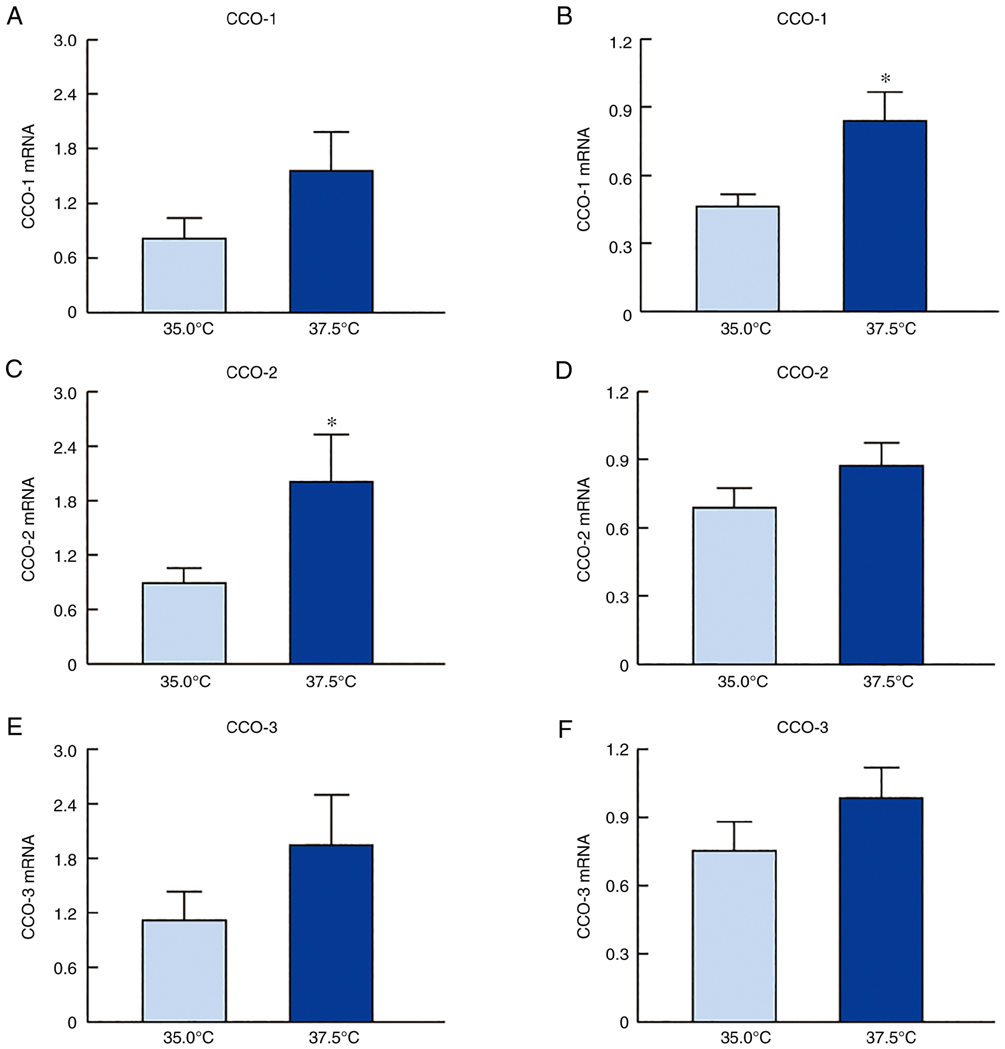
CCO mRNA levels in SRA01/04 or iHLEC-NY2 cells under low- or high-temperature condition. Expression of CCO-1 mRNA in (A) SRA01/04 cells and (B) iHLEC-NY2 cells at 35.0°C or 37.5°C. Expression of CCO-2 mRNA in (C) SRA01/04 cells and (D) iHLEC-NY2 cells at 35.0°C or 37.5°C. Expression of CCO-3 mRNA in (E) SRA01/04 cells and (F) iHLEC-NY2 cells at 35.0°C or 37.5°C. n=5. *P<0.05 vs. 35.0°C. CCO, cytochrome c oxidase; mRNA, messenger ribonucleic acid. CCO, Cytochrome c oxidase; iHLEC-NY2, immortalized human lens epithelial cells NY2.

**Figure 3.**
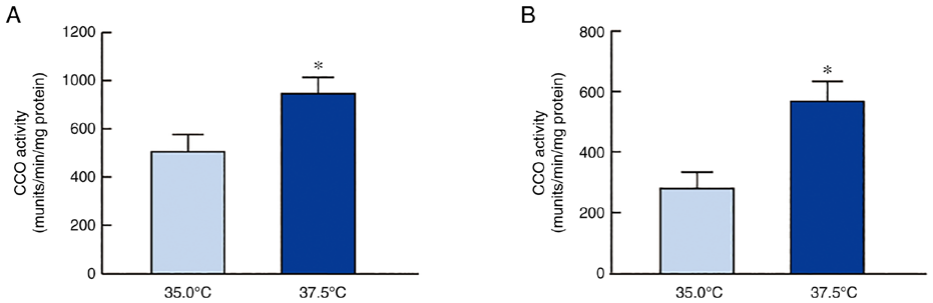
CCO activity of (A) SRA01/04 cells and (B) iHLEC-NY2 cells at 35.0°C or 37.5°C. n=3. *P<0.05 vs. 35.0°C. CCO, cytochrome c oxidase. CCO, Cytochrome c oxidase; iHLEC-NY2, immortalized human lens epithelial cells NY2.

Fig. 2 shows the changes in the mRNA levels of CCO-1, CCO-2, and CCO-3 in the SRA01/04 and iHLEC-NY2 cells under low- and high-temperature cultures. The CCO mRNA expression levels in both cell lines were higher when the cells were cultured at 37.5°C than when cultured at 35.0°C. Particularly, the level of CCO-2 mRNA expression in SRA01/04 cells and that of CCO-1 in iHLEC-NY2 cells cultured under high-temperature conditions (37.5°C) was significantly higher than those in cells cultured under low-temperature conditions (35.0°C). In addition, CCO activity in SRA01/04 and iHLEC-NY2 cells was measured under low- and high-temperature conditions (Fig. 3). CCO activity was enhanced following high-temperature culture (37.5°C), with the activity in SRA01/04 and iHLEC-NY2 showing values 1.48- and 2.02-fold higher than those at 35.0°C (P<0.05), respectively.

Mitochondria are closely related to apoptosis. We used immortalized cells, and cell proliferation was slightly slower at 35.0°C than at 37.5°C; however, the cells still proliferated at 35.0°C. There were a few abnormalities in the cell morphology (Fig. 1A-D). Because few abnormal cells were observed, we considered that the difference in temperature between 37.5°C and 35.0°C had almost no effect on apoptosis. To corroborate this prediction, we evaluated apoptosis using an apoptosis detection kit with Annexin V as an indicator. Almost no apoptotic cells were labeled with Annexin V among the two cell lines (Fig. S1).

### ATP production in SRA01/04 and iHLEC-NY2 cells under high-temperature conditions

**Figure 4.**
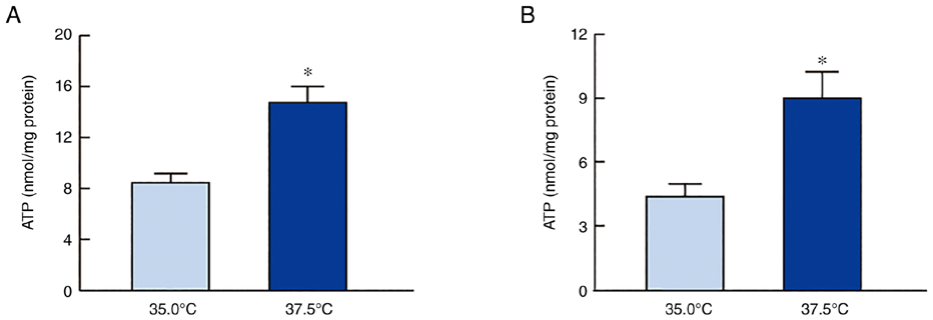
ATP levels in (A) SRA01/04 cells and (B) iHLEC-NY2 cells at 35.0°C or 37.5°C. n=3. *P<0.05 vs. 35.0°C. ATP, adenosine triphosphate; iHLEC-NY2, immortalized human lens epithelial cells NY2.

**Figure 5.**
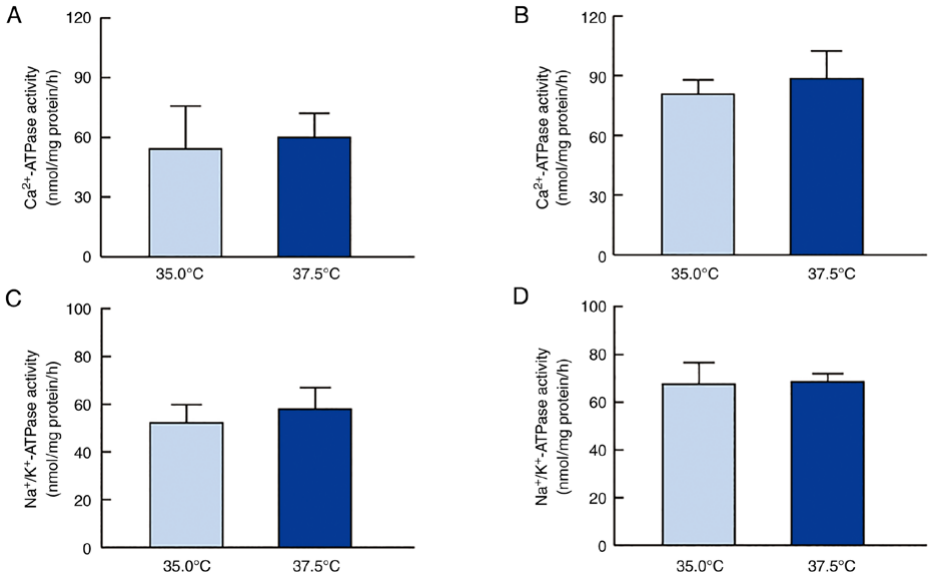
ATPase activities of SRA01/04 and iHLEC-NY2 cells under low- or high-temperature condition. Changes in Ca^2+^-ATPase activity of (A) SRA01/04 cells and (B) iHLEC-NY2 cells at 35.0°C or 37.5°C. Changes in Na^+^/K^+^-ATPase activity of (C) SRA01/04 cells and (D) iHLEC-NY2 cells at 35.0°C or 37.5°C. n=3. ATPase, adenosine triphosphatase; iHLEC-NY2, immortalized human lens epithelial cells NY2.

Fig. 4 shows the effects of low- and high-temperature culture on ATP levels in SRA01/04 and iHLEC-NY2 cells. The high temperature increased the ATP levels in SRA01/04 and iHLEC-NY2 cells. The ATP levels in SRA01/04 cells cultured at 37.5°C were 1.74-fold higher than those in cells cultured at 35.0°C (P<0.05). In addition, ATP levels in iHLEC-NY2 cells were significantly higher than those in cells cultured at 35.0°C. ATP is consumed by ATPase, and the ion balance is regulated in HLE cells. Therefore, we investigated the changes in Ca^2+^-ATPase and Na^+^/K^+^-ATPase activity in SRA01/04 and iHLEC-NY2 cells cultured at low or high temperature (Fig. 5). Fig. 5A and 5B show the effect of high temperature on Ca^2+^-ATPase activity in HLE cells. Ca^2+^-ATPase activity did not significantly differ between HLE cells cultured at 35.0°C or 37.5°C. Fig. 5C and 5D show the changes in Na^+^/K^+^-ATPase activity in SRA01/04 and iHLEC-NY2 cells cultured at 35.0°C or 37.5°C. As observed for Ca^2+^-ATPase activity, Na^+^/K^+^-ATPase activity was similar in HLE cells cultured at 37.5 and 35.0°C.

## Discussion

The effects of global warming have become a major concern worldwide. The global mean surface air temperatures in 2081-2100 are estimated to be 2.6-4.8°C higher than those in 1986–2005 if greenhouse gas emissions continue to increase on a high trajectory (RCP 8.5), as predicted by the Representative Concentration Pathways (RCPs) (27). RCPs were divided into four scenarios [RCP 2.6 (low), 4.5, 6.0, and 8.5 (high)]. The estimated temperature of the crystalline lens is affected by the ambient temperature (surface temperature) around the eye and core temperature of the body. Therefore, in our previous study (11), simulation analysis using a supercomputer based on the biothermal transport equation was performed in a model with an eye tissue resolution of 1 mm based on the Japanese body model developed by the National Institute of Information and Communications Technology (28). The results showed that simulated lens temperatures ranged from 35.0 to 37.5°C at ambient eye temperatures of 19 to 35°C in a typical environment. In the present study, cellular experiments were conducted at 35.0 and 37.5°C from the perspective of lens temperature *in vivo*. We previously showed that the estimated temperature of the crystalline lens is greatly affected by the ambient temperature (surface temperature) around the eye as well as the core temperature of the body. We performed computer simulation analysis using a supercomputer based on the biothermal transport equation, which showed that the cumulative thermal dose estimation in the crystalline lens was highly correlated with nuclear cataract prevalence.

An increased cell density via HLE cell proliferation is the underlying cause of nuclear cataract (13). Moreover, our previous epidemiological study showed that the risk of nuclear cataract development is significantly higher in high-temperature environments (9,10). In this study, we investigated the relationship between temperature and cell proliferation via mitochondrial function, which is a nuclear cataract-inducing risk factor, using HLE cell lines (SRA01/04 and iHLEC-NY2). We found that expression of the mitochondrial genome (CCO1-3) was enhanced at high temperature, resulting in sufficient ATP content and cell proliferation. A previous report comparing the same conditions as in this study, 35.0 and 37.5°C, reported slower mitotic cell division in mouse preimplantation embryos at lower temperatures; this division was accelerated as the temperature was increased from 35.0 to 37.5°C (29). Thus, the same phenomenon was observed in the lens as in other tissues.

SRA01/04 is a HLE cell line that has been used in several studies worldwide. The SRA01/04 cell line was derived from lens epithelial cells isolated and cultured from the lens of 12 infants with retinopathy during prematurity. A large T antigen from simian vacuolating virus 40 (SV40) was inserted downstream of the Rous sarcoma virus promoter and transfected into the cultured lens epithelial cells using the pGEM3Zf plasmid vector to establish the immortalized HLE cell line SRA01/04 (21).

The iHLEC-NY2 cell line was derived from the lens epithelial cells of primary culture (30) of a single adult transparent HLE cell. A plasmid vector designed to specifically introduce the modified SV40 large T antigen-GFP (mSV40-GFP) into the pseudo-attachment (att) P site was prepared. Cultured crystalline lens epithelial cells were transfected by micro-electroporation and cloned to establish the immortalized human crystalline lens epithelial cell line iHLEC-NY2 (21).

The difference between SRA01/04 and iHLEC-NY2 cells is the site at which the immortalizing gene was inserted. Because the SV40 gene was randomly introduced into SRA01/04 cells, it is possible that multiple SV40 genes were introduced. As a result, SV40 may have been introduced into a functional gene region of the cell. In contrast, in iHLEC-NY2 cells, the SV40 gene was specifically introduced only into the attP site, which does not have a function, thus ensuring that the original gene sequence of the cells was undisturbed. Crystallin proteins expressed in HLE cells have been detected and differentiated into lens fiber cells by changing the medium composition (31).

First, we confirmed that the proliferation of SRA01/04 and iHLEC-NY2 cells was indeed enhanced in high-temperature culture (Fig. 1). Thereafter, we demonstrated the effects of high-temperature culture on mitochondrial function by monitoring the expression level and activity CCO enzyme. This enzyme is the terminal enzyme of the mitochondrial respiratory chain, which reduces oxygen to water and pumps protons across the inner mitochondrial membrane (19), thus playing an important role in ATP production. CCO contains 13 subunits per monomer with the mitochondrial genome coding for the largest three catalytic subunits, whereas 10 subunits, namely 4, 5a, b, 6a, b, c, 7a, b, c and 8, are coded by nuclear DNA (32,33). The three mitochondrial subunits are downregulated earlier and to a greater extent than the nuclear subunits in response to functional inactivation (19). Thus, changes in the expression of CCO isoforms are related to CCO activity. We measured the CCO activity in this study and changes in the mRNA expression of the mitochondrial subunits (CCO1-3) under high-temperature culture and found that their expression was increased (Fig. 2). The CCO mRNA expression levels in both cell lines were higher when the cells were cultured at 37.5°C than when cultured at 35.0°C (Fig. 2). Specifically, the level of CCO-2 mRNA expression in SRA01/04 cells and that of CCO-1 in iHLEC-NY2 cells cultured under high-temperature conditions (37.5°C) was significantly higher than that in cells cultured under low-temperature conditions (35.0°C) (Fig. 3). In addition, it is necessary to measure the changes in CCO protein or activity to corroborate the mRNA data. Therefore, we measured CCO activity, which was enhanced under high-temperature culture (Fig. 3). These results suggest that the mitochondrial respiratory pathway in HLE cells was accelerated under high-temperature conditions. In contrast, the distinct roles of CCO-1, CCO-2, and CCO-3 in the lens tissue are unclear. In addition, the other 10 subunits (nuclear subunit 4, 5a, b, 6a, b, c, 7a, b, c and 8) may affect the enhanced CCO activity under high-temperature conditions. However, it is not yet clear whether other factors (mitochondrial membrane potential, glycolytic rate, and OXPHOS rate) are involved. Further studies are needed to clarify this mechanism.

Next, changes in the ATP content in the HLE cells were measured (Fig. 4). High-temperature culture enhanced ATP levels in both SRA01/04 and iHLEC-NY2 cells. These data support that mitochondrial function was enhanced, as shown in Figs. 2 and 3. Ca^2+^-ATPase plays a central role in Ca^2+^ transport and the maintenance of low internal Ca^2+^ concentrations using intracellular ATP. Further, Na^+^/K^+^-ATPase distributes ions between the intracellular and extracellular spaces and is responsible for total-body sodium homeostasis. Therefore, when evaluating ATP production at different temperatures, it is important to investigate whether these generalized ATPase activities are altered, as the ATPases consume ATP. However, no significant difference was observed between different HLE cells and temperature conditions (Fig. 5). We found that neither Ca^2+^-ATPase nor Na^+^/K^+^-ATPase activity was involved in regulating ATP levels in HLE cells cultured at 37.5 and 35.0°C; however, further studies are needed to determine whether other ATPases are involved in the ion balance, such as proton-ATPase (H^+^-ATPase).

**Figure 6.**
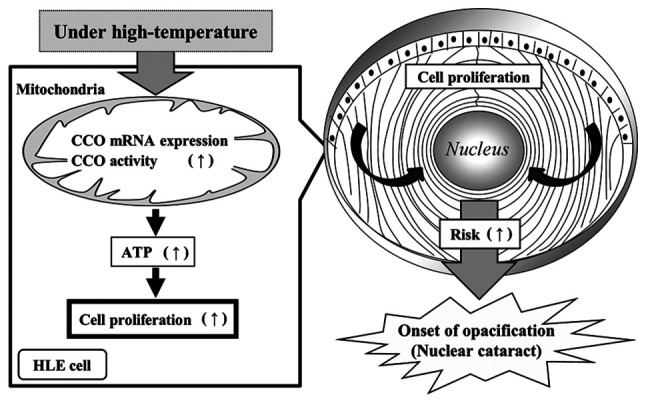
Schematic/graphic diagram depicting relationships between mitochondrial function and cell proliferation in the HLE cell under high-temperature condition. CCO, cytochrome c oxidase; mRNA, messenger ribonucleic acid; ATP, adenosine triphosphate; HLE cell, human lens epithelium cell.

It has been reported that the enhancement of density via HLE cell proliferation is the underlying cause of nuclear cataracts (13) and that sufficient ATP via mitochondrial function promotes cell proliferation (14). Taken together, we hypothesized that the enhanced ATP levels were related to cell proliferation. However, studies using knockdown or overexpression of CCO are needed to clarify this hypothesis. The CCO inhibition observed in this study has been reported to be reversible, and mitochondrial CCOs are more susceptible to stimulation (19,31,32). In addition, many studies reported that mitochondrial mutations in CCO cause disease (34,35). Accordingly, we focused on CCOs to evaluate the change in mitochondrial function. We showed that the mitochondrial function and ATP content in HLE cells were enhanced under high-temperature conditions. Therefore, we hypothesized that high-temperature conditions increased the ATP content by activating mitochondrial function and accelerating HLE cell proliferation. Enhanced cell proliferation may lead to a higher cell density in the central anterior capsule, resulting in an increased risk of nuclear cataract development (Fig. 6). Unlike other tissues in the body, the crystalline lens does not contain capillaries; thus, substances involved in nutrient supply or metabolism such as ions and amino acids move inside the lens cortex using the intercellular adhesion molecules of the lens fiber cells or through cell influx such as Na^+^/K^+^ ATPase activity (36). Because the periphery of the lens is surrounded by the lens capsule, which mainly contains type-4 collagen, cell proliferation and high cell density may affect the internal hydrostatic pressure of the lens, resulting in a decrease in nutrient supply and metabolites to the lens nucleus. The glycolytic and oxidative phosphorylation rates should also be determined to measure the mitochondrial membrane potential, which would reveal changes in mitochondrial function. Although there are reports on the involvement of ATP production and cell division, there are no data on the oxidative phosphorylation rate (37,38), which will be evaluated in our future studies.

It is important to perform similar experiments after HLE cells are exposed to ultraviolet rays, which is another risk factor for cataract formation (10,39). Additionally, in animal experiments, as the effect occurs on the lens in the living eye, the temperature may be affected by the aqueous humor and blood perfusion. Moreover, further studies are required to determine the precise mechanisms of cell proliferation and ATP levels in HLE cells. Therefore, we will investigate the effects of rearing animals under different environment temperatures on the lens. In addition, we are currently investigating the changes in ATP production and cell proliferation in the lens of patients living in subtropical regions (i.e., Mkuranga, Tanzania; Singapore; Amami, Kagoshima, Japan; and Sanya, Hainan, China), temperate regions (Monzen, Ishikawa, Japan; Taiyuan, Shanxi, China; and Shenyang, Liaoning, China), and subarctic regions (Reykjavik, Iceland). Another limitation of this study was that the estimated lens temperature simulated from the ambient temperature around the eye and body temperature was set as the culture medium temperature (35.0°C or 37.5°C); however, the influences of other temperatures on the cells were not verified and should be further investigated. Second, in an experiment using normal diploid cells (MRC-5), the authors reported that the cells could be passaged 57.2 times when at 37.0°C, but only 29.2 times at 40°C, indicating that increasing the temperature to above 40°C affects cell proliferation (40). Experiments conducted under high-temperature conditions of 37.5°C or higher have not been reported, and we identified the upper limit for the high-temperature condition as 37.5°C. Third, measurement of CCO-1-3 protein by western blotting is important for clarifying the relationships of the mitochondrial genome. However, we did not perform this experiment. Instead, we show both the mRNA level and activity of CCO. Western blotting will be performed in our further studies.

In conclusion, the expression and activity of CCO in the mitochondrial genome were enhanced under high-temperature culture, resulting in sufficient ATP content and cell proliferation. High cell density via HLE cell proliferation is the underlying cause of nuclear cataracts (13). Hence, high ATP production via mitochondrial activation may contribute to HLE proliferation, resulting in lens opacity. These findings support the results of our previous epidemiological study on the relationship between the prevalence of nuclear cataracts and elevated environmental temperatures (9,10).

## Supplementary Material

Supporting Data

## Data Availability

The datasets used and/or analyzed during the current study are available from the corresponding author on reasonable request.

## Abbreviations

ATP: adenosine-5′-triphosphate
CCO: cytochrome c oxidase
DMEM: Dulbecco's modified Eagle's medium
FBS: fetal bovine serum
HEPES: 4-(2-hydroxyethyl)piperazine-1-ethanesulfonic acid
HLE: human lens epithelial
iHLEC-NY2: immortalized human lens epithelial cells NY2
RCP: representative concentration pathway
Tris-HCl: tris(hydroxymethyl)aminomethane hydrochloride

## References

[1] World Health Organization (WHO) (2021). Vision 2020: The RIGHT TO SIGHT. Global initiative for the elimination of avoidable blindness. https://www.who.int/blindness/Vision2020_report.pdf.

[2] Moreau KL, King JA (2012). Protein misfolding and aggregation in cataract disease and prospects for prevention. Trends Mol Med.

[3] Shenoy AM, Kothadia AD, Shabaraya AR, Rajan MS, Viradia UM, Patel NH (2011). Evaluation of cataract preventive action of phycocyanin. Int J Pharm Sci Drug Res.

[4] GBD 2019 Blindness and Vision Impairment Collaborators and Vision Loss Expert Group of the Global Burden of Disease: Study (2021). Causes of blindness and vision impairment in 2020 and trends over 30 years, and prevalence of avoidable blindness in relation to VISION 2020: The right to sight: An analysis for the global burden of disease study. Lancet Glob Health.

[5] Vashist P, Talwar B, Gogoi M, Maraini G, Camparini M, Ravindran RD, Murthy GV, Fitzpatrick KE, John N, Chakravarthy U (2011). Prevalence of cataract in an older population in India: the India study of age-related eye disease. Ophthalmology.

[6] Klein BE, Klein R, Lee KE (2002). Incidence of age-related cataract over a 10-year interval: The Beaver dam eye study. Ophthalmology.

[7] Lee J, Kim MJ, Tchah H (2008). Higher-order aberrations induced by nuclear cataract. J Cataract Refract Surg.

[8] Miranda MN (1979). The geographic factor in the onset of presbyopia. Trans Am Ophthalmol Soc.

[9] Sasaki K, Sasaki H, Jonasson F, Kojima M, Cheng HM (2004). Racial differences of lens transparency properties with aging and prevalence of age-related cataract applying a WHO classification system. Ophthalmic Res.

[10] Miyashita H, Hatsusaka N, Shibuya E, Mita N, Yamazaki M, Shibata T, Ishida H, Ukai Y, Kubo E, Sasaki H (2019). Association between ultraviolet radiation exposure dose and cataract in Han people living in China and Taiwan: A cross-sectional study. PLoS One.

[11] Kodera S, Hirata A, Miura F, Rashed EA, Hatsusaka N, Yamamoto N, Kubo E, Sasaki H (2020). Model-based approach for analyzing prevalence of nuclear cataracts in elderly residents. Comput Biol Med.

[12] Ayaki M, Ohde H, Yokoyama N (1993). Size of the lens nucleus separated by hydrodissection. Ophthalmic Surg.

[13] Liu X, Liu Y, Zheng J, Huang Q, Zheng H (2000). Lens epithelial cell proliferation and cell density in human age-related cataract. Yan Ke Xue Bao.

[14] Yan XJ, Yu X, Wang XP, Jiang JF, Yuan ZY, Lu X, Lei F, Xing DM (2017). Mitochondria play an important role in the cell proliferation suppressing activity of berberine. Sci Rep.

[15] Sun N, Youle RJ, Finkel T (2016). The mitochondrial basis of aging. Mol Cell.

[16] Kauppila TES, Kauppila JHK, Larsson NG (2017). Mammalian mitochondria and aging: An update. Cell Metab.

[17] Sebastian D, Palacin M, Zorzano A (2017). Mitochondrial dynamics: Coupling mitochondrial fitness with healthy aging. Trends Mol Med.

[18] Lenka N, Vijayasarathy C, Mullick J, Avadhani NG (1998). Structural organization and transcription regulation of nuclear genes encoding the mammalian cytochrome c oxidase complex. Prog Nucleic Acid Res Mol Biol.

[19] Liang HL, Ongwijitwat S, Wong-Riley MT (2006). Bigenomic functional regulation of all 13 cytochrome c oxidase subunit transcripts in rat neurons in vitro and in vivo. Neuroscience.

[20] Nagai N, Mano Y, Otake H, Shibata T, Kubo E, Sasaki H (2019). Changes in mitochondrial cytochrome c oxidase mRNA levels with cataract severity in lens epithelia of Japanese patients. Mol Med Rep.

[21] Ibaraki N, Chen SC, Lin LR, Okamoto H, Pipas JM, Reddy VN (1998). Human lens epithelial cell line. Exp Eye Res.

[22] Yamamoto N, Takeda S, Hatsusaka N, Hiramatsu N, Nagai N, Deguchi S, Nakazawa Y, Takata T, Kodera S, Hirata A (2020). Effect of a lens protein in low-temperature culture of novel immortalized human lens epithelial cells (iHLEC-NY2). Cells.

[23] Sasaki H, Jonasson F, Shui YB, Kojima M, Ono M, Katoh N, Cheng HM, Takahashi N, Sasaki K (2002). High prevalence of nuclear cataract in the population of tropical and subtropical areas. Dev Ophthalmol.

[24] Nagai N, Ito Y (2012). Dysfunction in cytochrome c oxidase caused by excessive nitric oxide in human lens epithelial cells stimulated with interferon-gamma and lipopolysaccharide. Curr Eye Res.

[25] Nagai N, Ito Y (2007). Adverse effects of excessive nitric oxide on cytochrome c oxidase in lenses of hereditary cataract UPL rats. Toxicology.

[26] Nagai N, Liu Y, Fukuhata T, Ito Y (2006). Inhibitors of inducible nitric oxide synthase prevent damage to human lens epithelial cells induced by interferon-gamma and lipopolysaccharide. Biol Pharm Bull.

[27] Stocker TF, Qin D, Plattner GK, Tignor M, Allen SK, Boschung J, Nauels A, Xia Y, Bex V, Midgley PM, IPCC (Intergovernmental Panel on Climate Change): Summary for policymakers (2013). Climate Change 2013: The Physical Science Basis, Contribution of Working Group I to the Fifth Assessment Report of the Intergovernmental Panel on Climate Change.

[28] Nagaoka T, Watanabe S, Sakurai K, Kunieda E, Watanabe S, Taki M, Yamanaka Y (2004). Development of realistic high-resolution whole-body voxel models of Japanese adult males and females of average height and weight, and application of models to radio-frequency electromagnetic-field dosimetry. Phys Med Biol.

[29] Walters EA, Brown JL, Krisher R, Voelkel S, Swain JE (2020). Impact of a controlled culture temperature gradient on mouse embryo development and morphokinetics. Reprod Biomed Online.

[30] Yamamoto N, Kato Y, Sato A, Hiramatsu N, Yamashita H, Ohkuma M, Miyachi EI, Horiguchi M, Hirano K, Kojima H (2016). Establishment of a new immortalized human corneal epithelial cell line (iHCE-NY1) for use in evaluating eye irritancy by in vitro test methods. In Vitro Cell Dev Biol Anim.

[31] Hiramatsu N, Nagai N, Kondo M, Imaizumi K, Sasaki H, Yamamoto N (2021). Morphological comparison between three-dimensional structure of immortalized human lens epithelial cells and Soemmering's ring. Med Mol Morphol.

[32] Kadenbach B, Jarausch J, Hartmann R, Merle P (1983). Separation of mammalian cytochrome c oxidase into 13 polypeptides by a sodium dodecyl sulfate-gel electrophoretic procedure. Anal Biochem.

[33] Kuhn-Nentwig L, Kadenbach B (1985). Isolation and properties of cytochrome c oxidase from rat liver and quantification of immunological differences between isozymes from various rat tissues with subunit-specific antisera. Eur J Biochem.

[34] Afkhami E, Hheidari MM, Khatami M, Ghadamyari F, Dianatpour S (2020). Detection of novel mitochondrial mutations in cytochrome c oxidase subunit 1 (COX1) in patients with familial adenomatous polyposis (FAP). Clin Transl Oncol.

[35] Wangpermtam P, Petmitr S, Punyarit P, Klongnoi B, Sanguansin S (2019). Down-regulation of mitochondrial NADH and cytochrome b gene associated with high tumor stages in head and neck squamous cell carcinoma. Arch Oral Biol.

[36] Nakazawa Y, Donaldson PJ, Petrova RS (2019). Verification and spatial mapping of TRPV1 and TRPV4 expression in the embryonic and adult mouse lens. Exp Eye Res.

[37] McMillan SN, Scarff CA (2022). Cryo-electron microscopy analysis of myosin at work and at rest. Curr Opin Struct Biol.

[38] Maeshima K, Matsuda T, Shindo Y, Imamura H, Tamura S, Imai R, Kawakami S, Nagashima R, Soga T, Noji H (2018). A transient rise in free Mg^2+^ ions released from ATP-Mg hydrolysis contributes to mitotic chromosome condensation. Curr Biol.

[39] Yoshitomi Y, Osada H, Satake H, Kojima M, Saito-Takatsuji H, Ikeda T, Yoshitake Y, Ishigaki Y, Kubo E, Sasaki H, Yonekura H (2019). Ultraviolet B-induced Otx2 expression in lens epithelial cells promotes epithelial-mesenchymal transition. Biol Open.

[40] Thompson KV, Holliday R (1973). Effect of temperature on the longevity of human fibroblasts in culture. Exp Cell Res.

