# Supplementary material for: Function of mitochondrial cytochrome c oxidase is enhanced in human lens epithelial cells at high temperatures
Source: Mol Med Rep. 2022 Dec 2;27(1):19. doi: 10.3892/mmr.2022.12906 (PMC9743390; doi:10.3892/mmr.2022.12906)

Figure S1. Results of apoptosis analysis using flow cytometry. SRA01/04 cell at (A) 35.0˚C showed 0.28% early apoptosis and 0.47% late apoptosis and at (B) 37.5˚C showed 0.47% early apoptosis and 0.56% late apoptosis. iHLEC-NY2 cell at (C) 35.0˚C showed 0.05% early apoptosis and 0.05% late apoptosis and at (D) 37.5˚C showed 0.09% early apoptosis and 0.08% late apoptosis. GFP, green fluorescent protein; APC, allophycocyanin; iHLEC-NY2, immortalized human lens epithelial cells NY2.

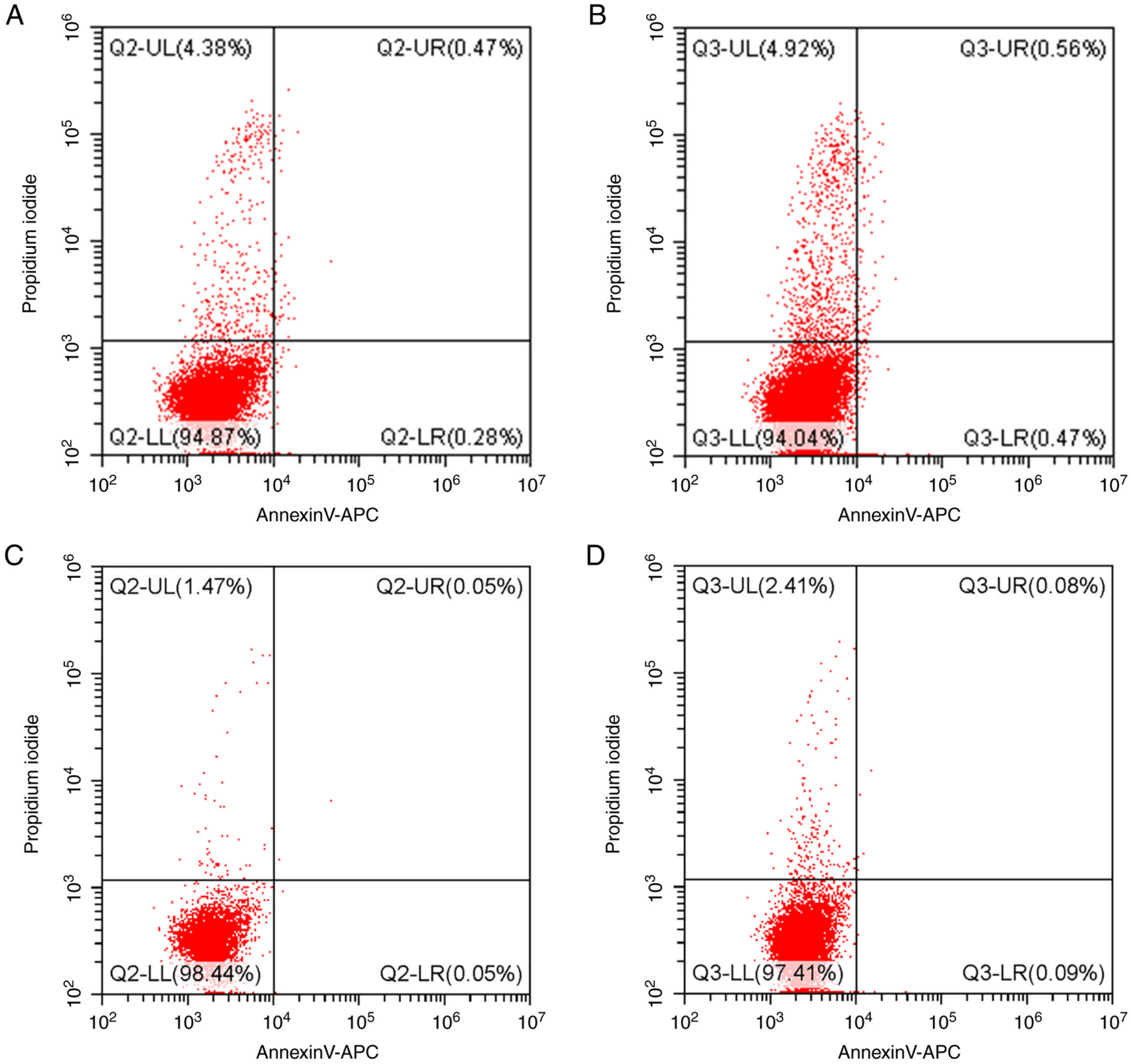

Supplement: Supporting Data [file Supplementary_Data.pdf]
